# Engineered repressors are potent inhibitors of androgen receptor activity

**DOI:** 10.18632/oncotarget.1360

**Published:** 2014-01-21

**Authors:** Greg N. Brooke, Sue M. Powell, Derek N. Lavery, Jonathan Waxman, Laki Buluwela, Simak Ali, Charlotte L. Bevan

**Affiliations:** ^1^ Department of Surgery and Cancer, Imperial Centre for Translational and Experimental Medicine, Imperial College London, W12 0NN, UK; ^2^ School of Biological Sciences, University of Essex, Wivenhoe Park, Colchester CO4 3SQ, UK

**Keywords:** androgen receptor, prostate cancer, anti-androgen, castrate resistant prostate cancer

## Abstract

Prostate cancer growth is dependent upon the Androgen Receptor (AR) pathway, hence therapies for this disease often target this signalling axis. Such therapies are successful in the majority of patients but invariably fail after a median of 2 years and tumours progress to a castrate resistant stage (CRPC). Much evidence exists to suggest that the AR remains key to CRPC growth and hence remains a valid therapeutic target. Here we describe a novel method to inhibit AR activity, consisting of an interaction motif, that binds to the AR ligand-binding domain, fused to repression domains. These ‘engineered repressors’ are potent inhibitors of AR activity and prostate cancer cell growth and importantly inhibit the AR under circumstances in which conventional therapies would be predicted to fail, such as AR mutation and altered cofactor levels.

## INTRODUCTION

The Androgen Receptor (AR) is a ligand-dependent transcription factor and like other members of the Nuclear Receptor family, has a modular structure consisting of the N-terminal domain, the central DNA-binding domain and the C-terminal ligand-binding domain [1]. The receptor contains two activation functions (AF), the predominant AF-1 in the N-terminus and the weaker ligand dependent AF-2 in the ligand binding domain (LBD) [2]. In the absence of ligand the receptor is localised in the cytoplasm, associated with a heat shock protein complex that holds the receptor in a ligand binding competent conformation. Upon ligand binding the receptor undergoes a conformational change, which promotes nuclear localisation, dimerization, recruitment of accessory proteins and an intramolecular N-/C-terminal interaction [3]. The N-/C-terminal interaction is important in reducing ligand off rate and increasing receptor stability [4]. The importance of this interaction in AR transcriptional activity, however, appears to be promoter specific [5, 6].

Coactivators are proteins that bind to and enhance the activity of transcription factors while not directly binding DNA themselves. Steroid receptor coactivators, such as the p160 family, often interact with the AF-2 surface of agonist-bound nuclear receptors, via an α-helical LxxLL motif [7, 8]. However, the AR differs from other steroid receptors in that it has greater affinity for phenylalanine rich motifs, such as the FQNLF motif found in the N-terminus of the AR that mediates the N-/C-terminal interaction, and those found in coactivators such as ARA70 [9-11]. This difference in interaction motif preference between steroid receptors is a result of a deeper coactivator interaction groove on the surface of the active (holo) form of the AR LBD, which can accommodate the bulkier phenylalanine residues [10, 12]. Conversely AR, like other steroid receptors, can interact with corepressor proteins (such as NCoR) in the presence of antagonists, which promote a different LBD conformation [13-15]. Corepressors bind to the LBD of nuclear receptors at a distinct region that nevertheless overlaps with the coactivator-interacting region and subsequently inhibit transcription of target genes [13, 16].

Prostate cancer growth is almost always dependent upon the androgen-signalling axis and as a result, androgen deprivation therapy is a common treatment option for this disease. This involves one or both of chemical castration to ablate circulating androgens and antiandrogens – steroidal or non-steroidal ligands that bind to the AR LBD but do not promote subsequent target gene activation, either due to simple competition, by preventing receptor dimerization and/or nuclear translocation and DNA-binding, by promoting formation of a repressive complex at the androgen response element (ARE), or a mixture of mechanisms [15, 17]. Although antiandrogens are initially successful in the majority of patients, relapse is inevitable and the tumours progress to a “castrate resistant” stage (CRPC), for which few therapeutic options exist. Much evidence exists to suggest that the AR is still driving growth, even in the androgen-depleted environment, including frequent AR amplification and mutation, as well as alterations in cofactor levels and activities [18]. The AR therefore remains a valid target for CRPC and novel methods to inhibit the AR are required. Ideally such therapies would be active even in circumstances where current therapies would be predicted to fail (e.g. mutations of the AR resulting in broadened ligand preference and promiscuous activation [11]).

Due to the limited efficacy of current antiandrogens, novel methods to inhibit AR signalling are being devised that directly target different regions of the AR. Zhang *et al*., for example, demonstrated that ectopic expression of a decoy androgen response element was successful at reducing AR activity [19]. Other studies demonstrated that targeting either the AF-1 or AF-2 of the AR using peptides, can successfully inhibit AR activity [20-22]. These consist of specific AR-binding motifs, which subsequently block crucial interactions such as the N-/C-terminal interaction and recruitment of coactivators. Another approach is the engineering of AR-specific corepressors. Reeb *et al.* performed a yeast 2-hybrid peptide screen against the full-length AR in the presence of the antiandrogen hydroxyflutamide [23]. Fusion of the lead interacting peptide with a silencing domain generated an AR corepressor with receptor specific inhibitory effects.

Here we describe the design and validation of AR engineered repressors that combine the desirable characteristics of coactivators and corepressors, in that they interact with the AR when it is in a holo conformation and block its activity. These consist of an interaction motif containing an FxxLF motif, fused to potent repression domains. Importantly, we demonstrate that these factors are successful in inhibiting the AR in circumstances thought to lead to castrate resistant prostate cancer.

## RESULTS

### Engineered repressor design

**Figure 1:**
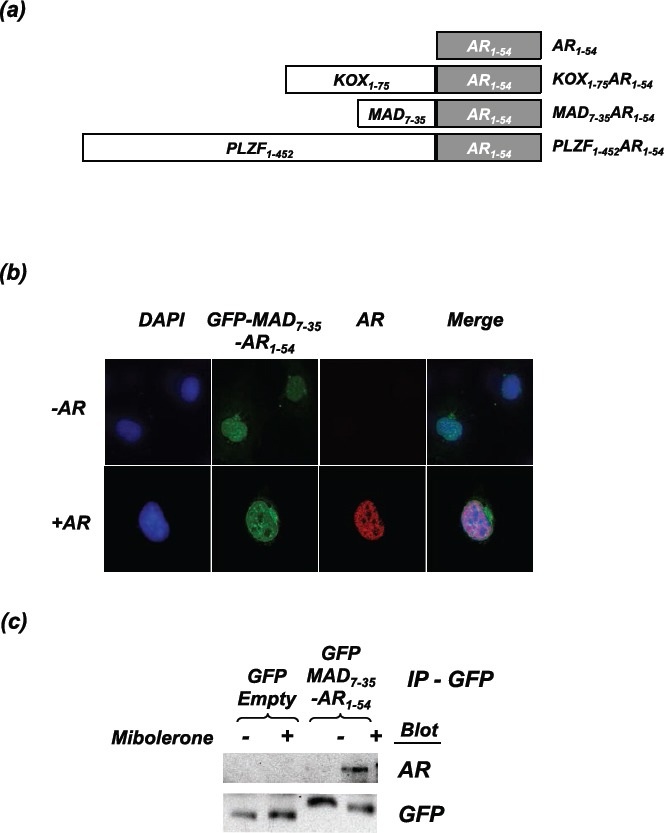
The repressor constructs enter the nucleus and interact with the active androgen receptor **(a)** Schematic representation of the engineered repressors (not drawn to scale). **(b)** COS-1 cells were transfected with the AR and GFP-MAD_7-35_-AR_1-54_. Cells were fixed following 2hrs of treatment with mibolerone. Confocal microscopy was used to visualise the localisation of GFP-MAD_7-35_-AR_1-54_ (green) and the full-length AR (stained using ALEXA 594 (red)). Nuclear staining = DAPI (blue). **(c)** COS-1 cells were transfected with the AR and and GFP-MAD_7-35_-AR_1-54_ or GFP-Empty. Cells were treated ± mibolerone for 2hrs and complexes immunoprecipitated with an anti-GFP antibody. Immunoprecipitated complexes were separated using SDS-PAGE and immunoblotted for AR (using an antibody that does not recognise residues 1-54) and GFP.

Previous studies have demonstrated that peptides designed to target intra- and inter-receptor interactions can successfully inhibit AR activity [20, 21]. For example, peptides consisting of an FxxLF α-helix, which can bind to AF-2 of the AR, inhibit the N-/C-terminal interaction and reduce AR activity [21]. In an attempt to make a more potent inhibitor of the AR, we fused amino acids 1-54 of the AR, which contains the ^23^FQNLF^27^ motif known to interact with the AR LBD (termed the interaction motif), to known repression domains from different proteins: MAD (amino acids 7-35 [24]), KOX (amino acids 1-75 [25]) and PLZF (amino acids 1-452 [26]). The resulting constructs are MAD_7-35_-AR_1-54_, KOX_1-75_-AR_1-54_, PLZF_1-452_-AR_1-54_ (Figure 1a). These repressors should not only sterically disrupt coactivator binding and the N-/C-terminal interaction, but also bring a potent repression domain in close proximity to the receptor upon activation by ligand.

### The engineered repressors interact with the active Androgen Receptor

As proof of principle to confirm that the repressors and the AR interact, MAD_7-35_-AR_1-54_ was fused to GFP and co-transfected into COS-1 cells with an AR expression vector. Confocal microscopy demonstrated that MAD_7-35_-AR_1-54_ is predominantly nuclear and appears to colocalise with the agonist bound AR (Figure 1b), suggesting that the proteins interact. This interaction was confirmed using co-immunoprecipitation, whereby a GFP antibody (against the MAD_7-35_-AR_1-54_ construct) also pulled-down full-length AR (Figure 1c). Importantly, this interaction was ligand-dependent, as would be expected since the interaction of ^23^FQNLF^27^ within AR_1-54_ with the AR ligand binding domain is dependent upon AF-2 being in an active conformation [27].

### The engineered repressors inhibit Androgen Receptor activity

**Figure 2:**
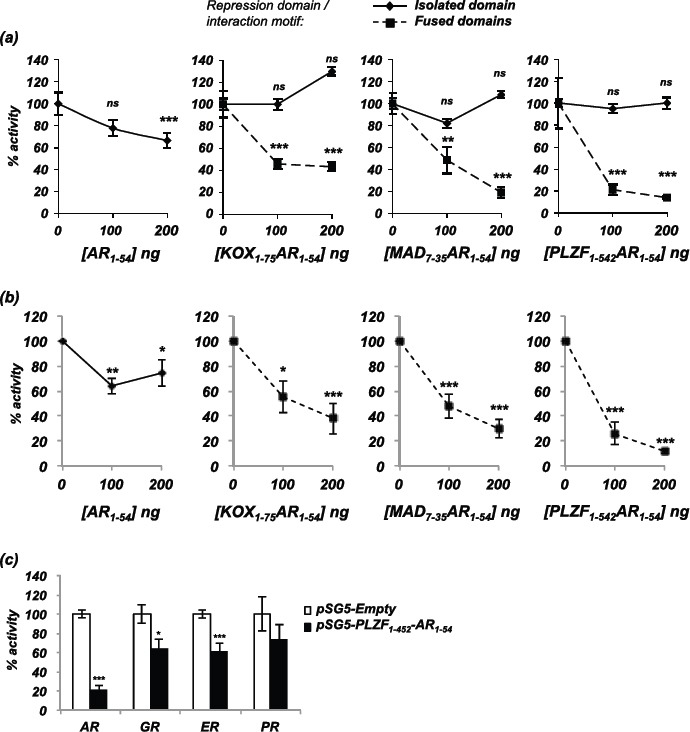
Inhibition of AR activity by the engineered repressors **(a)** COS-1 cells were transiently transfected with vectors for the AR, TAT-GRE-E1B-LUC, PDM-LACZ-β-GAL and the repressors or interaction motif either in isolation (solid line) or as a fusion (broken line). **(b)** PC3-WTAR were transiently transfected with vectors for TAT-GRE-E1B-LUC, PDM-LACZ-β-GAL, AR_1-54_ or the repressors. **(c)** COS-1 cells were transfected with vectors encoding the androgen, glucocorticoid, oestrogen or progesterone receptors (AR, GR, ER, PR), the respective luciferase reporter construct (TAT-GRE-E1B-LUC or ERE-LUC), PDM-LACZ-β-GAL and PLZF_1-452_-AR_1-54_. Luciferase activity was normalised with β-galactosidase expression and results expressed as a % of AR activity in the absence of the repressors. Mean of 3 independent duplicates ±1SE. T-Test - **p<0.005, ***p<0.0005

To investigate the repressive activity of the engineered repressors compared to the interaction motif and repression domains in isolation, each was transfected into COS-1 cells along with an AR expression plasmid and an androgen-responsive luciferase reporter gene. The N-terminal 54 amino acid fragment of AR expressed in isolation reduced AR activity by 34% (Figure 2a). Repression domains in isolation had no effect on AR activity (Figure 2a, solid lines), but when fused to AR_1-54_ the resulting fusion constructs had greater inhibitory action than the interaction motif alone: maximal repression for AR_1-54_- KOX_1-75_ was 57%, for MAD_7-35_-AR_1-54_ was 81% and for PLZF_1-452_-AR_1-54_ was 86% (Figure 2a, broken lines). To ensure that this effect was not an artefact of cell line used or transiently transfected AR, PC3-WTAR cells (PC3 prostate cancer cell line stably expressing AR [28]) were transfected with a luciferase reporter and the repressors. Similar to the repressive effects demonstrated in the COS-1 cell line, the engineered repressors potently inhibited AR activity in PC3 cells (Figure 2b).

The interaction domain utilised in the engineered repressors contains a phenylalanine rich motif (FQNLF), which should be specific for the AR. To investigate if the other closely related steroid receptors (glucocorticoid, oestrogen and progesterone receptors) were also inhibited by the engineered repressors, transcription assays were performed using these receptors. COS-1 cells were therefore transfected with plasmids encoding PLZF_1-452_-AR_1-54_ and the steroid receptors, and cells treated with the corresponding ligand. The PLZF_1-452_-AR_1-54_ repressor weakly inhibited glucocorticoid, oestrogen and progesterone receptor activity by <40% compared to approximately 80% inhibition of the AR (Figure 2c).

**Figure 3:**
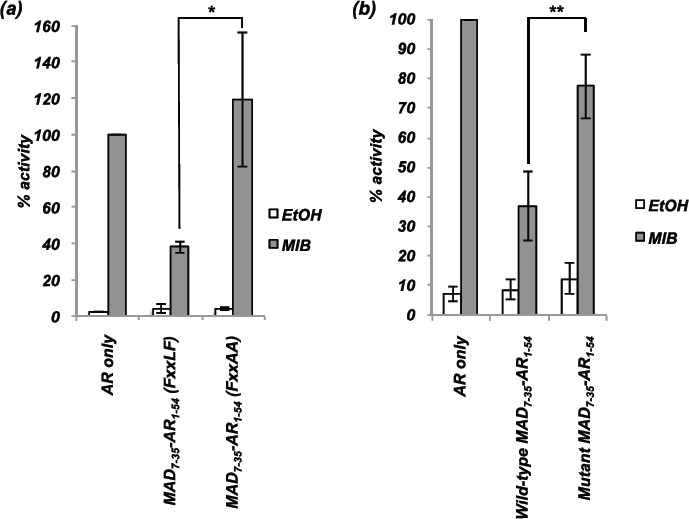
The repression domain and interaction motif are both essential for maximal repression of the AR COS-1 cells were transiently transfected with vectors for the AR, TAT-GRE-E1B-LUC, PDM-LACZ-β-GAL and MAD_7-35_-AR_1-54_ with wild-type or mutated **(a)** interaction motif (FQNLF to FQNAA) or **(b)** repression domain (L12P and A16P). Mean ±1SE. T-Test - ** p<0.005, *** p<0.0005.

To confirm that both the interaction domain and repression domain were required for inhibitory action, point mutations were introduced into these domains and activity measured using transcription assays. Mutation of the FQNLF interaction motif to FQNAA abolished repressive activity of MAD_7-35_-AR_1-54_ (Figure 3a). To confirm that the repression domain also contributes to repressive potential, mutations were introduced in to the repression motif of the MAD_7-35_AR_1-54_ vector. Mutation of residues L12 and A16 to prolines disrupted intrinsic transrepression activity (Supplementary Figure 1 and [24]). Fusion of the mutated MAD_7-35_ repression domain with AR_1-54_ generated a repressor with markedly weaker repressive activity compared to wild-type MAD_7-35_AR_1-54_ (Figure 3b). We therefore conclude that fusion of an interaction motif with a repression domain generates a potent repressor of AR activity and that both domains are essential for maximal inhibitory potential.

### The engineered repressors successfully block Androgen Receptor activity in circumstances that promote hormone therapy failure

**Figure 4:**
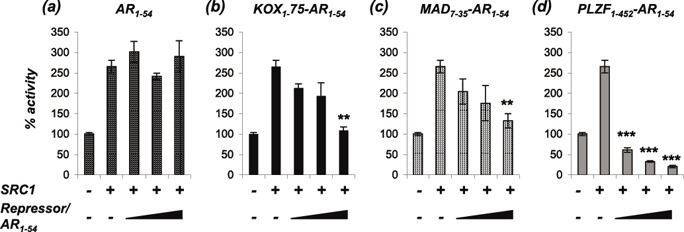
The repressors block AR activity enhanced by SRC1 COS-1 cells were transiently transfected with vectors for the AR, TAT-GRE-E1B-LUC, PDM-LACZ-β-GAL, SRC1 and **(a)** AR_1-54_, **(b)** KOX_1-75_-AR_1-54_, **(c)** MAD_7-35_-AR_1-54_ or **(d)** PLZF_1-542_-AR_1-54_. Luciferase data was normalised to β-galactosidase expression and expressed as a % of AR activity in the absence of cofactor. Mean ±1SE. T-Test - ** p<0.005, *** p<0.0005.

Much evidence exists to suggest that the AR can drive CRPC growth. One possible mechanism to explain therapy failure leading to CRPC is alterations in levels and/or activities of AR co-regulators sensitising the pathway to low levels or weakly androgenic ligands. Indeed, Gregory *et al*., demonstrated that the coactivators SRC1 (Steroid Receptor Coactivator 1) and TIF2 (transcriptional intermediary factor 2) were over-expressed in the majority of CRPCs [29], and altered expression and localisation of AR corepressors in prostate cancer has also been described (for example [30-34]). To determine whether the engineered repressors can inhibit AR activity enhanced by coactivator over-expression, COS1 cells were transfected with expression plasmids for the AR, SRC1 and increasing amounts of engineered repressor. SRC1 enhanced AR activity by 2.5-fold and the LBD interaction motif (AR_1-54_) was unable to compete with this enhanced activity (Figure 4a). By contrast, the engineered repressors were all able to significantly reduce the SRC1-enhanced AR activity (Figure 4b-d).

**Figure 5:**
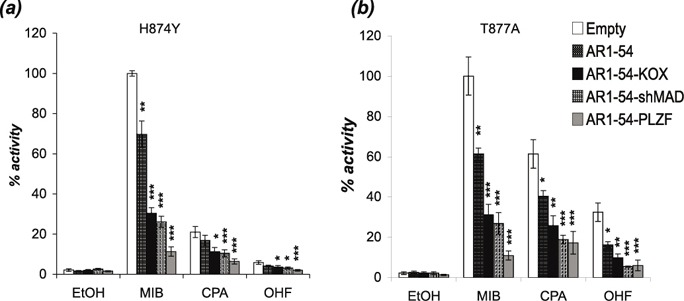
The engineered repressors block mutant ARs activated by androgen and antiandrogens COS-1 cells were transiently transfected with vectors for the **(a)** H874Y or **(b)** T877A mutant ARs, TAT-GRE-E1B-LUC, PDM-LACZ-β-GAL and the repressors or interaction motif. EtOH – ethanol, MIB - mibolerone, CPA - cyproterone acetate, OHF - hydroxyflutamide. Mean ±1SE. T-Test * p<0.05, ** p<0.005, ***p<0.0005.

Mutations in the AR gene are rare in the early stages of prostate cancer, but their frequency increases significantly in advanced stages of the disease [35, 36]. For example, Gaddipati *et al.* found the T877A substitution in 25% of metastatic PCa samples analysed [37]. Some mutations appear to offer a growth advantage by reducing ligand specificity, allowing non-androgen ligands such as the antiandrogens cyproterone acetate (CPA) and hydroxyflutamide (OHF) to activate the receptor. To investigate if the engineered repressors can inhibit mutant ARs activated by these antiandrogens, COS-1 cells were transfected with two AR mutants commonly associated with prostate cancer (H874Y and T877A) and the engineered repressors. AR mutants H874Y and T877A were activated by mibolerone and to a lesser extent by CPA and OHF (Figure 5a and b). The engineered repressors were considerably more potent inhibitors of these AR mutants than AR_1-54_ for all ligand conditions.

### The engineered repressors inhibit prostate cancer cell growth

**Figure 6:**
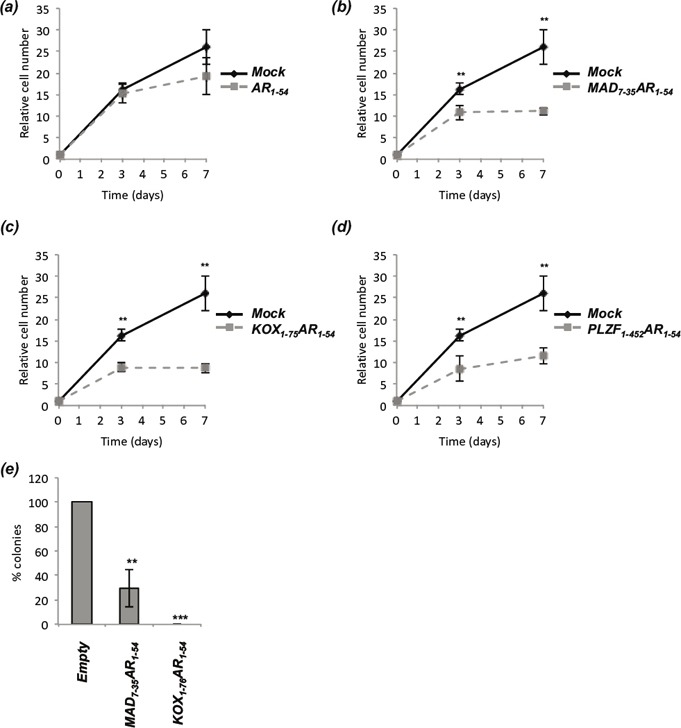
The engineered repressors significantly reduce prostate cancer proliferation LNCaP cells were transiently co-transfected with vectors for GFP and **(a)** AR_1-54_, **(b)** MAD_7-35_AR_1-54_, **(c)** KOX_1-75_AR_1-54_ or **(d)** PLZF_1-452_AR_1-54_. GFP positive cells were counted 24hrs after transfection (set as day 0), and subsequently counted on days 3 and 7. Automated counting (ImageJ) was performed on 10 random fields of view on an Axiovert Fluorescent Microscope (Zeiss), repeated in triplicate and representative figures given. **(e)** LNCaP-C42 cells were transiently transfected with vectors encoding MAD_7-35_-AR_1-54_, KOX_1-75_-AR_1-54_ or Empty and the Zeocin selection cassette. Cells were selected with Zeocin for 3 weeks, colonies fixed, stained using crystal violet and colony number quantified. Mean ± 1SE. T-Test *p<0.05, ** p<0.005, *** p<0.0005.

We have demonstrated that the engineered repressors are potent inhibitors of AR activity and inhibit receptor signalling in circumstances that would be predicted to promote CRPC. To determine whether these inhibitory effects translate into inhibition of prostate cancer growth, LNCaP cells were co-transfected with plasmids encoding GFP and the engineered repressors, and GFP positive cells counted over a time-course (Figure 6 and Supplementary Figure 2). AR_1-54_ had little effect upon proliferation (Figure 6a), whereas the fusion constructs all significantly inhibited LNCaP growth within 3 days (Figure 6b-d). At day 7, MAD_7-35_-AR_1-54_, KOX_1-75_-AR_1-54_ and PLZF_1-452_-AR_1-54_ inhibited LNCaP growth by 67, 57 and 55% respectively.

Colony formation assays were performed using the more aggressive LNCaP-C42 prostate cancer cell line [38] and the 2 shorter repressors (MAD_7-35_-AR_1-54_, KOX_1-75_-AR_1-54_). Exogenous expression of MAD_7-35_-AR_1-54_ and KOX_1-75_-AR_1-54_ significantly reduced colony formation, with no colonies emerging in any experiment using the latter construct (Figure 6E).

## DISCUSSION

Hormone therapy targeting the AR pathway, is a common therapeutic option for the treatment of PCa. Although initially successful in the majority of patients, such therapies invariably fail after a median of 2 years and the tumours progress to the Castrate Resistant (CRPC) stage. Few therapeutic options exist for this stage of the disease, but much evidence exists to suggest that the AR continues to drive CRPC growth, and hence remains a valid therapeutic target [39].

Several groups have previously demonstrated that peptides designed to bind to regions important in AR activity (e.g. Activation Functions 1 and 2) can successfully inhibit AR activity [20-23]. We reasoned that fusing such peptides to repression domains derived from transcriptional repressor proteins would yield more potent inhibitors of AR activity. Therefore, we generated engineered repressors consisting of an AR LBD interaction motif (AR_1-54_), fused to known repression domains from a number of potent transcriptional repressors, namely KOX, MAD and PLZF [24-26]). These are uniquely designed to interact with the AR when it is in an active conformation and subsequently block transcriptional activity. Reporter assays demonstrated that fusion of the repression domains to the AR_1-54_ interaction motif generated potent repressors of AR activity. Mutation of the interaction motif (FQNLF to FQNAA) completely abolished repression, whereas mutation of the MAD_7-35_ repression domain significantly reduced, but did not abolish inhibitory potential, likely as a result of the intact interaction motif since this alone possesses some inhibitory activity ([40] and data herein).

Upon ligand binding, steroid receptors interact with coactivators, which enhance transcriptional activity [8]. Despite high homology between the LBDs of steroid receptors, the AR is idiosyncratic in that it not only interacts with α-helical LxxLL motifs present in coactivators such as SRC1 but also interacts with, and has preference for, similar phenylalanine rich motifs, e.g. FxxLF motifs, found in the coactivator ARA70 and in the N-terminus of the AR itself [41, 42]. To provide AR selectivity of our repressors, we therefore utilised an N-terminal fragment of the AR (AR_1-54_) containing the ^23^FQNLF^27^ motif that mediates the N-/C-terminal interaction [42]. The resulting PLZF_1-452_-AR_1-54_ repressor was found to be more specific for the AR but did also weakly inhibit the other steroid receptors. Previous studies using phenylalanine-rich motifs have also found weak cross-reactivity with these steroid receptors [21], hence refinement of such motifs warrants further investigation.

Multiple mechanisms have been described to explain therapy failure and CRPC, including alterations in AR cofactor levels/localisation. Transcriptional competition assays showed that, while the interaction motif alone was unable to compete with the effects of increased SRC1 expression, all of the engineered repressors were able to successfully inhibit AR activity under these conditions.

Mutation of the AR is another mechanism believed to contribute to CRPC [35]. Identified mutations in prostate cancer cluster in the LBD and several have been demonstrated to reduce ligand specificity. For example, the prostate cancer associated AR mutations H874Y and T877A can be activated by non-androgenic hormones (e.g. oestradiol and progesterone) and antiandrogens (e.g. CPA and OHF) [11]. Importantly, the engineered repressors were able to reduce the activity of these mutants irrespective of the activating ligand, successfully blocking the receptors when activated by both androgen and anti-androgens.

We have therefore demonstrated that fusion of repression domains with an AR-specific interaction motif generates potent inhibitors of the AR that are more potent than interaction motifs alone, and that these constructs are active in circumstances where conventional therapeutics would be predicted to fail. Growth assays demonstrated that these repressive effects upon the AR do translate to growth inhibition of prostate cancer cells. Therefore, the use of engineered repressors is a potential therapeutic approach to inhibit CRPC, and methods to refine and deliver such constructs, for example viral delivery or nanoparticles, merit further investigation.

## METHODS

### Ligands

Mibolerone was purchased from Perkin Elmer (MA, USA). Oestradiol, Progesterone, Dexamethasone and Cyproterone Acetate were from Sigma Aldrich (MO, USA) and Bicalutamide and Hydroxyflutamide from Toronto Research Chemicals (Toronto, Canada).

### Constructs

Amino acids 1-54 of the AR were PCR amplified and cloned into pSG5 using *Xho*I and *Bam*HI. Repression domains from KOX (a.a. 1-75), MAD (a.a. 7-35) and PLZF (a.a. 1-452) were subsequently PCR cloned upstream of AR_1-54_ using *Eco*RI and *Xho*I. To generate the pCDNA4.0 (Invitrogen, Paisley, UK) engineered repressor constructs, the repressors were PCR cloned into the *Kpn*I and *Xba*I sites. Site directed mutagenesis (QuickChange II, Agilent DE, USA) was performed on the pSG5-MAD_7-35_AR_1-54_ construct to generate constructs with mutation of the AR_1-54_ domain (FxxLF to FxxAA) or mutation of the repression domain (L12P and A16P). All constructs were confirmed by diagnostic digest and sequencing. The following plasmids have been previously described: pSG5-SRC1e [43], pSVAR [44], TAT-GRE-EIB-LUC [45].

### Cell Culture

COS1 and LNCaP cells were obtained from ATCC and grown at 37°C 5% CO_2_. COS1 cells were grown in Dulbecco's modified Eagle's medium (DMEM) and LNCaP/LNCaP-C42 cells in Roswell Park Memorial Institute (RPMI) medium 1640 (Life technologies, Strathclyde, UK), both supplemented with 2mM L-Glutamine, 100units/ml penicillin, 100mg/ml streptomycin (PSG) and 10% foetal bovine serum. PC3-WTAR cells [28] were grown as above with the addition of 4μg/ml of Geneticin (Life Technologies, Gaithersburg, MD). For experiments involving hormone manipulation, cells were cultured in phenol red free DMEM or RPMI supplemented with PSG and 5% charcoal stripped foetal bovine serum (stripped medium).

### Confocal Microscopy

COS1 cells were plated at 30% confluence on cover slips in 24 well plates. Cells were transfected with pSVAR and pGFP-MAD_7-35_AR_1-54_ using FuGENE 6 (Promega, WI, USA) and incubated for 24hrs before treatment with ligand for 2hrs. Wells were washed 3x with PBS and incubated in 1% paraformaldehyde for 10 min, washed 3x in PBS and incubated for a further 10 min in 0.1% Triton X-100 in PBS. Wells were again washed 3x with PBS, incubated in blocking solution (5% BSA in PBS) for 30 min and a further 1 hr with an AR antibody (AR C19, Santa Cruz Biotechnology, TX, USA) diluted 1:200 in blocking solution. Wells were washed 3x with PBS, re-blocked and incubated for 1hr with 594-Alexa Fluor conjugated secondary antibody (Invitrogen, Paisley, UK). A final 3x PBS washes was performed before the coverslips were mounted onto glass slides (Vectorshield containing DAPI, DAKO, Cambridge, UK). Images were obtained using a Zeiss Confocal Microscope, as previously described [46].

### Immunoprecipitation

COS1 cells were seeded at 70% confluence in 10cm plates in ‘stripped medium’ and transfected with plasmids encoding the AR and GFP-MAD_7-35_AR_1-54_ using FuGENE 6.0 (Promega, WI, USA). Cells were left for 24hrs, treated for 2hrs ± 10nM Mibolerone and lysed in IP buffer (150mM NaCl, 1% NP-40, 50mM Tris pH8.0, 1mM DTT) containing freshly added protease inhibitors. Lysates were spun at 13,000 rpm (15 min, 4°C), supernatants transferred to fresh tubes and protein concentration measured using the DC protein assay (BioRad, CA, USA). Lysates were pre-cleared with 50 μl of sepharose beads (Sigma-Aldrich, MO, USA, 30min of rotation at 4°C), supernatant transferred to fresh tubes and incubated with anti-GFP antibody (Abcam, Cambridge, UK) for 1hr with rotation before addition of 50 μl of sepharose beads. After 1hr, beads were washed 3 times with IP buffer, laemmli loading buffer added to the beads and samples boiled before protein separation using immunoblotting, as previously described [30].

### Reporter Assays

COS1 cells were transfected using the calcium phosphate method as previously described [11]. Cells were seeded in 24 well plates and transfected per well with steroid receptor expression plasmids (100 ng), pDM-LACZ-β-GAL (100 ng), 1μg TAT-GRE-E1B-LUC/ERE-LUC (1 μg) and 0-200ng of pSG5-AR_1-54_, pSG5-MAD_7-35_-AR_1-54_, KOX_1-75_-AR_1-54_, PLZF_1-452_-AR_1-54_, pSG5-MAD_7-35_-AR_1-54_(FQNAA)or pSG5-MAD_7-35_(L12P, A16P)-AR_1-54_. PC3-WTAR cells were transfected with 50ng PDM-LACZ-β-GAL, 250ng TAT-GRE-E1B-LUC and 0-200ng of pSG5-AR_1-54_, pSG5-MAD_7-35_-AR_1-54_, KOX_1-75_-AR_1-54_, PLZF_1-452_-AR_1-54_ using FuGENE HD (Promega, WI, USA) following the manufacturer's instructions. Twenty-four hours after transfection, cells were treated with 10nM of ligand and cells incubated for a further 16 hrs. Luciferase and β-galactosidase expression was measured as previously described [11].

### Growth Assays

LNCaP cells were plated to 50% confluence in 6 well plates in ‘stripped media’ containing 10nM Mibolerone and co-transfected with pGFP-Empty and plasmids encoding AR_1-54_, MAD_7-35_-AR_1-54_, KOX_1-75_-AR_1-54_ or PLZF_1-452_-AR_1-54_ using JetPrime (VWR International Ltd., Leicestershire, UK), following the manufacturers instructions. 24hrs after transfection (set as day 0), GFP positive cells were visualised in 10 random fields of view from triplicate wells using an Axiovert Fluorescent Microscope (Zeiss, x20 objective) and the number of GFP positive cells assessed using Image J (NIH). Subsequent cell counting was performed on days 3 and 7.

### Colony Formation Assay

Colony formation assays were based on Kawano *et al.* [47]. Briefly, LNCaP-C42 cells were plated in 6 well plates and transfected with 2 μg of plasmid using JetPrime (VWR International Ltd., Leicestershire, UK). Twenty-four hours after transfection, cells were trypsinised and seeded in 10cm dishes. RPMI media containing Zeocin (300μg/ml, Invitrogen, Paisley, UK) was changed twice weekly and cells grown for 3 weeks, following which cells were washed in PBS, fixed and stained in 0.2% (w/v) crystal violet, 20% MeOH (10min, RT). Plates were washed 6 times in ddH_2_O and cells counted manually.

## SUPPLEMENTARY FIGURES


