# Supplementary material for: Engineered repressors are potent inhibitors of androgen receptor activity
Source: Oncotarget. 2014 Jan 21;5(4):959–69. doi: 10.18632/oncotarget.1360 (PMC4011597; doi:10.18632/oncotarget.1360)
Supplement: Supplementary file 1 [file oncotarget-05-959-s001.pdf]

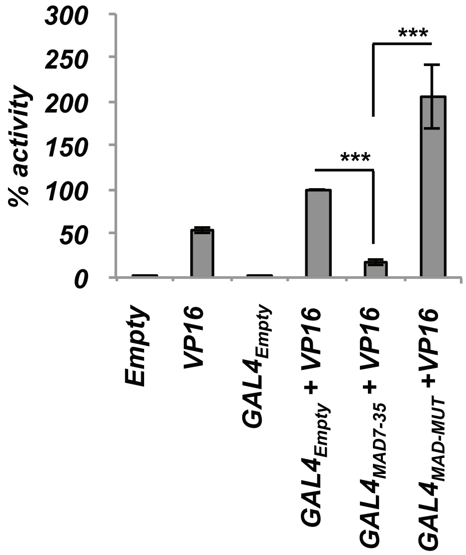

Supplementary Figure 1: Mutation of the MAD_{7-35} domain reduces repressive activity. Transrepression assays were performed in COS1 cells. Cells were transfected with plasmids encoding a luciferase reporter regulated by upstream LEXA and GAL4 binding sites, Empty/MAD_{7-35}/MAD MUT fused to GAL4 and VP16 fused to LEXA. Mean ± 1SE. T-Test *** $p<0.0005$.

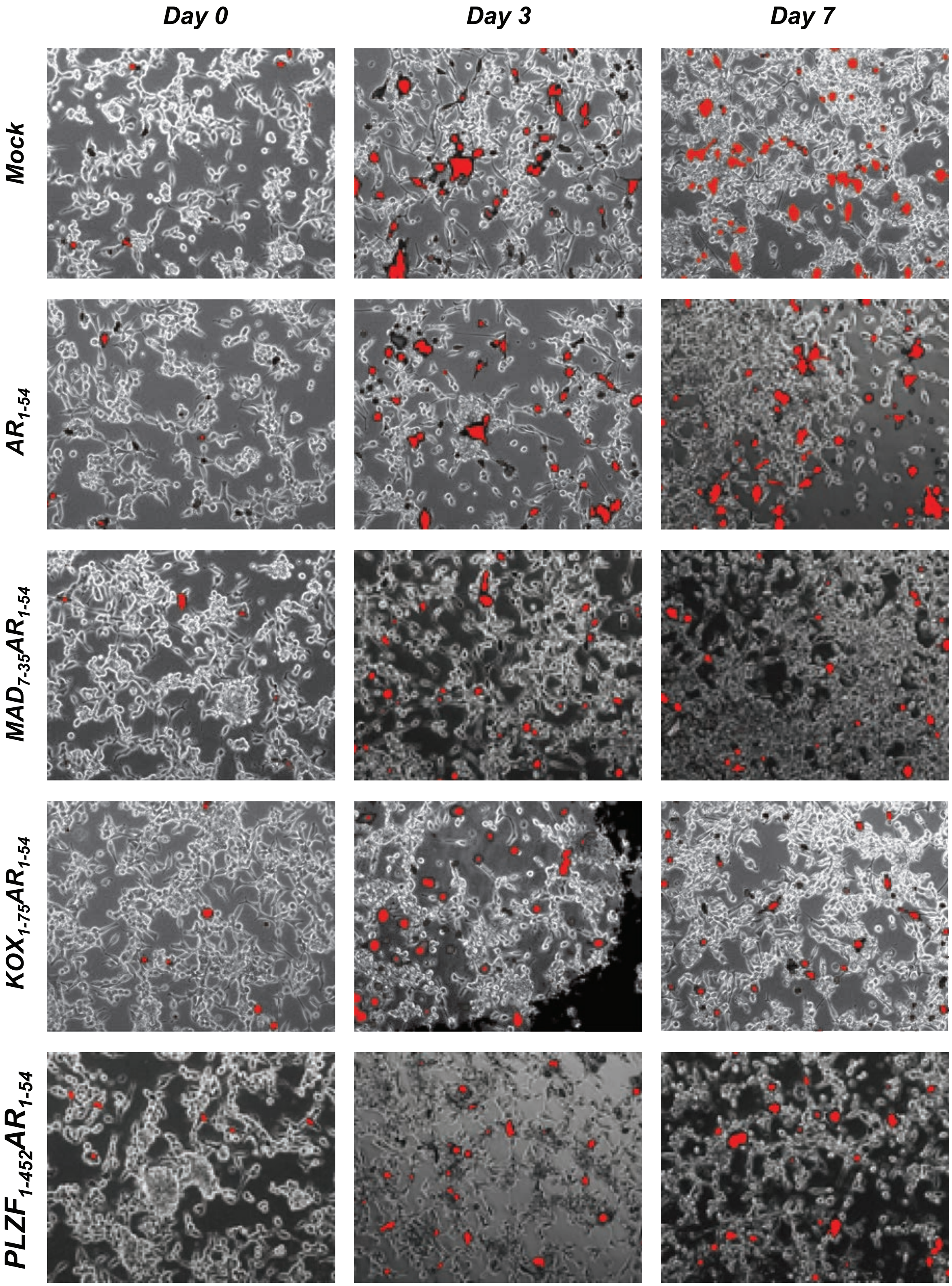

Supplementary Figure 2: The repressors significantly reduce LNCaP proliferation. LNCaP cells were transiently co-transfected with vectors for GFP and AR_{1-54}, $MAD_{7-35}AR_{1-54}$, $KOX_{1-75}AR_{1-54}$ or $PLZF_{1-452}AR_{1-54}$. The number of GFP positive cells (highlighted in red for clarity) was assessed after 24hrs (set as day 0) in 10 random fields of view on an Axiovert Fluorescent Microscope (Zeiss), repeated in triplicate and representative figures given. GFP positive cells were subsequently counted on days 2 and 6.
